# Anticancer Activity of MPT0E028, a Novel Potent Histone Deacetylase Inhibitor, in Human Colorectal Cancer HCT116 Cells *In Vitro* and *In Vivo*

**DOI:** 10.1371/journal.pone.0043645

**Published:** 2012-08-22

**Authors:** Han-Lin Huang, Hsueh-Yun Lee, An-Chi Tsai, Chieh-Yu Peng, Mei-Jung Lai, Jing-Chi Wang, Shiow-Lin Pan, Che-Ming Teng, Jing-Ping Liou

**Affiliations:** 1 Pharmacological Institute, College of Medicine, National Taiwan University, Taipei, Taiwan; 2 School of Pharmacy, College of Pharmacy, Taipei Medical University, Taipei, Taiwan; 3 Institute of Biotechnology and Pharmaceutical Research, National Health Research Institutes, Zhunan Town, Miaoli County, Taiwan; 4 Department of Pharmacology, Taipei Medical University, Taipei, Taiwan; 5 School of Chinese Medicine, College of Chinese Medicine, China Medical University, Taichung, Taiwan; Albert-Ludwigs-University, Germany

## Abstract

Recently, histone deacetylase (HDAC) inhibitors have emerged as a promising class of drugs for treatment of cancers, especially subcutaneous T-cell lymphoma. In this study, we demonstrated that MPT0E028, a novel *N*-hydroxyacrylamide-derived HDAC inhibitor, inhibited human colorectal cancer HCT116 cell growth *in vitro* and *in vivo*. The results of NCI-60 screening showed that MPT0E028 inhibited proliferation in both solid and hematological tumor cell lines at micromolar concentrations, and was especially potent in HCT116 cells. MPT0E028 had a stronger apoptotic activity and inhibited HDACs activity more potently than SAHA, the first therapeutic HDAC inhibitor proved by FDA. *In vivo* murine model, the growth of HCT116 tumor xenograft was delayed and inhibited after treatment with MPT0E028 in a dose-dependent manner. Based on *in vivo* study, MPT0E028 showed stronger anti-cancer efficacy than SAHA. No significant body weight difference or other adverse effects were observed in both MPT0E028-and SAHA-treated groups. Taken together, our results demonstrate that MPT0E028 has several properties and is potential as a promising anti-cancer therapeutic drug.

## Introduction

Histone deacetylase inhibitors (HDACi) are a promising new class of anticancer agents. HDACi inhibit tumor progression through their ability to regulate gene expression by promoting acetylation of histone and non-histone proteins [1], [2]. Currently, several HDACi, including SAHA, LBH589, PXD101, MS-275, and FK228, are being examined in clinical trials for their ability to treat various solid and hematological malignancies [3], [4]. The U.S. Food and Drug Administration (FDA) recently approved SAHA and FK228 for the treatment of cutaneous T-cell lymphoma [5]. HDAC inhibitors (HDACi) modulate the expression of several genes that regulate apoptosis, angiogenesis [6], [7], cell cycle progression, and cellular differentiation. They have minimal toxicity against normal cells [8]–[10]. Taken together, these findings are critical in designing target inhibitors of HDAC for the treatment of cancer and other diseases.

A close view of these clinical trials with small molecule HDACi indicated the hydroxamic acid or *N*-hydroxyacrylamide group play an important role in HDAC activity [11], [12]. We previously reported identification of the indoline-1-sulfonamide-containing compounds with apparent anticancer activity [13], [14]. On the basis of the observations above, we designed and synthesized a series of new class of histone deacetylase inhibitors. Among them, 3-(1-benzenesulfonyl-2,3-dihydro-1H-indol-5-yl)-*N*-hydroxy-acrylamide (MPT0E028) has the best inhibitory activity of HDACs. In this study, we examined the antitumor activities of MPT0E028 in several cancer cell lines from the NCI-60 cancer cell panel. We investigated the effects of MPT0E028 on cell cycle progression and apoptosis and explored possible molecular mechanisms that underlie its anticancer activity. In addition, we examined the effect of MPT0E028 on the growth of human colorectal cancer HCT116 cells *in vivo*, using a tumor xenograft model, which confirmed the antitumor effect of MPT0E028. Our results suggest that MPT0E028 is a promising therapeutic candidate for the treatment of human cancers.

**Figure 1:**
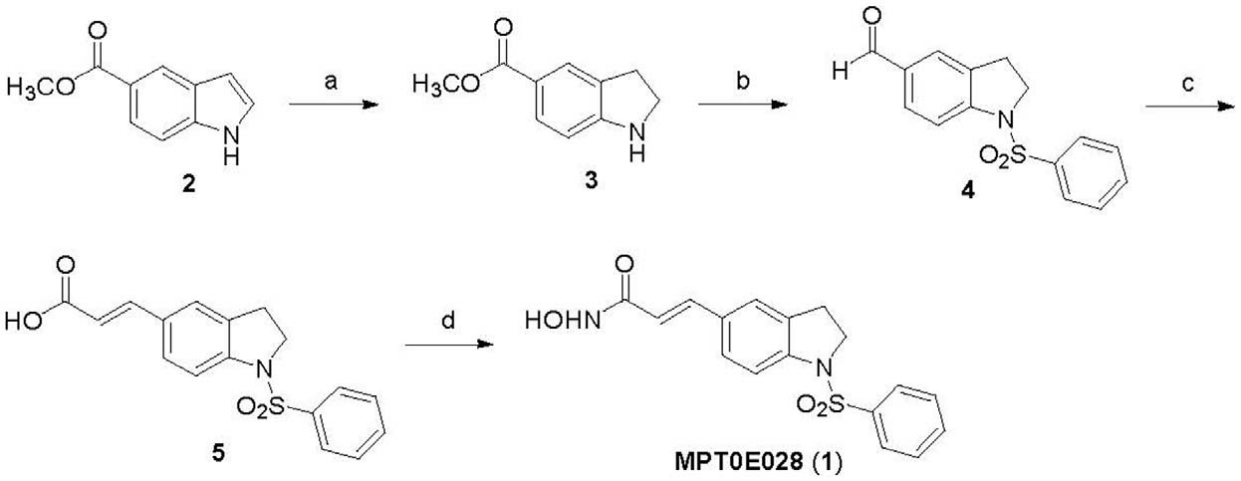
The synthesis of MPT0E028. Reagents and conditions. (a) NaBH_3_CN, AcOH, 0°C–r.t.; (b) (i) benzenesulfonyl chloride, pyridine, reflux; (ii) LiAlH_4_, THF, 0°C–r.t.; (iii) pyridinium dichromate (PDC), molecular sieves, CH_2_Cl_2_, r.t.; (c) (i) methyl(triphenylphosphorylidene)acetate, CH_2_Cl_2_, r.t.; (ii) 1 M LiOH_(aq)_, dioxane, 40°C; (d) (i) NH_2_OTHP, PYBOP, Et_3_N, DMF, r.t.; (iii) trifluoroacetic acid, CH_3_OH, r.t. Abbreviations: NaBH_3_CN, sodium cyanoborohydride; AcOH, acetyl acid; LiAlH_4_ (LAH), lithium aluminium hydride; THF, tetrahydrofuran; PDC, pyridinium dichromate; NH_2_OTHP, *O*-(tetrahydro-2H-pyran-2-yl)hydroxylamine; PYBOP, benzotriazol-1-yl-oxytripyrrolidinophosphonium hexafluorophosphate; Et_3_N, triethylamine; DMF, dimethylformamide; TFA, trifluoroacetic acid.

## Materials and Methods

### Materials

MPT0E028 and SAHA was synthesized by Dr. Jing-Ping Liou’s Lab. (School of Pharmacy, College of Pharmacy, Taipei Medical University, Taiwan), and the purity is more than 98% (Data S1). RPMI 1640, M199, fetal bovine serum (FBS), penicillin, streptomycin, and all other tissue culture reagents were obtained from Life Technologies (Grand Island, NY, USA). Antibodies against various proteins were listed as following: α-tubulin, PARP, actin, HRP-conjugated anti-mouse, and anti-rabbit IgG were from Santa Cruz (Santa Cruz, CA, USA); histone 3, actin, acetyl-µ-tubulin were from cell signaling were from Cell Signaling Technologies (Boston, MA, USA); caspase 3 was from Imgenex (San diego, CA, USA); acetyl-histone 3 was from Upstate Biotechnology (Lake Placid, NY, USA). Propidium iodide (PI), sulforhodamine B (SRB), and all of the other chemical reagents were obtained from Sigma Chemical (St. Louis, MO, USA).

**Table 1:** Anti-proliferative effects of MPT0E028 in the NCI-60 cell line panels.

Panels	Cell Line	GI_50_ (M)	TGI (M)	LC_50_ (M)	Panel	Cell Line	GI_50_ (M)	TGI (M)	LC_50_ (M)
**Leukemia**	CCRF-CEM	1.65E-7	>1.00E-4	>1.00E-4	**Melanoma**	LOX IMVE	2.22E-7	7.18E-7	2.88E-6
	HL-60 (TB)	4.39E-7	>1.00E-4	>1.00E-4		MALME-3M	3.75E-8	9.07E-7	>1.00E-4
	K-562	1.15E-7	>1.00E-4	>1.00E-4		M14	1.79E-7	1.11E-6	6.45E-6
	MOLT-4	3.17E-7	>1.00E-4	>1.00E-4		MDA-MB-435	1.46E-7	7.06E-7	1.13E-5
	RPMI-8226	8.11E-8	>1.00E-4	>1.00E-4		SK-MEL-2	4.33E-7	3.89E-6	3.49E-5
	SR	6.01E-8	>1.00E-4	>1.00E-4		SK-MEL-28	4.06E-7	2.74E-6	>1.00E-4
**Non-Small Cell Lung Cancer**	A549/ATCC	2.30E-7	1.09E-6	9.03E-6		SK-MEL-5	1.36E-7	3.10E-7	7.05E-7
	EKVX	5.34E-7	>1.00E-4	>1.00E-4		UACC-257	1.27E-7	1.65E-6	>1.00E-4
	HOP-62	1.87E-7	1.06E-6	1.92E-5		UACC-62	7.16E-8	3.39E-7	1.66E-6
	HOP-92	2.10E-7	8.14E-7	1.80E-5	**Ovarian Cancer**	IGROV1	1.83E-7	5.33E-7	2.39E-6
	NCI-H226	1.24E-6	3.88E-6	1.90E-5		OVCAR-3	2.33E-7	9.33E-7	7.30E-6
	NCI-H23	1.88E-7	9.22E-7	3.84E-6		OVCAR-4	5.09E-7	3.18E-5	>1.00E-4
	NCI-H322M	1.86E-7	1.14E-5	>1.00E-4		OVCAR-5	6.02E-8	3.16E-7	2.58E-6
	NCI-H460	1.44E-7	1.66E-6	2.16E-5		OVCAR-8	1.21E-7	1.75E-6	>1.00E-4
	NCI-H522	1.67E-7	8.45E-7	1.30E-5		NCI/ADR-RES	3.85E-8	2.39E-7	>1.00E-4
**Colon Cancer**	COLO 205	1.28E-7	2.64E-7	5.46E-7		SK-OV-3	1.90E-7	6.01E-7	4.26E-6
	HCC-2998	1.94E-7	5.22E-7	2.06E-6	**Renal Cancer**	786-0	3.00E-7	1.33E-6	4.02E-6
	HCT-116	5.63E-8	2.04E-7	5.66E-7		A498	2.43E-7	1.13E-6	5.13E-6
	HCT-15	4.30E-7	1.31E-5	9.02E-5		ACHN	1.67E-7	5.76E-7	4.08E-6
	HT29	1.18E-7	7.16E-7	6.81E-5		CAKI-1	2.18E-7	9.59E-7	>1.00E-4
	KM12	2.86E-7	1.49E-6	5.78E-6		RXF 393	1.12E-7	3.34E-7	9.98E-7
	SW-620	1.47E-7	1.12E-5	>1.00E-4		SN12C	4.58E-7	1.02E-5	>1.00E-4
**CNS Cancer**	SF-268	4.64E-7	3.40E-6	7.04E-5		TK-10	1.03E-7	6.60E-7	3.36E-5
	SF-295	6.03E-8	3.40E-7	2.43E-6		UO-31	1.69E-7	1.46E-6	5.63E-6
	SF-539	2.41E-7	1.15E-6	3.39E-6	**Prostate Cancer**	PC-3	2.40E-7	2.10E-5	>1.00E-4
	SNB-19	4.34E-7	1.64E-6	5.65E-6		DU-145	1.79E-7	1.92E-6	>1.00E-4
	SNB-75	5.91E-8	6.27E-6	3.28E-5	**Breast Cancer**	MCF-7	2.30E-7	1.05E-5	6.27E-5
	U251	1.99E-7	8.20E-7	3.76E-6		MAD-MB-231/ATCC	2.41E-7	8.22E-7	2.04E-5
						HS 578T	2.53E-7	1.79E-5	>1.00E-4
						BT-549	6.80E-7	5.84E-6	>1.00E-4
						T-47D	1.21E-7	4.45E-7	>1.00E-4
						MDA-MB-468	1.96E-7	1.15E-6	6.31E-6

GI_50_: 50% of growth inhibition;

TGI: total growth inhibition;

LC_50_: 50% of lethal concentration.

M: Concentration in molar (M).

### Cell Culture

The human colorectal cancer cell line HCT116, breast cancer cell line MDAMB231 and human umbilical vein endothelial cells (HUVEC) were purchased from American Type Culture Collection (ATCC; Manassas, VA). Ovarian cancer cell line NCI-ADR was obtained from the DTP Human Tumor Cell Line Screen (Developmental Therapeutics Program, NCI). HCT116, MDAMB231 and NCI-ADR cells were cultured in RPMI 1640 with 10% heat-inactivated fetal bovine serum (v/v) and penicillin (100 units/mL)/streptomycin (100 µg/mL). HUVEC was cultured in M199 with 20% heat-inactivated fetal bovine serum (v/v) and penicillin (100 units/mL)/streptomycin (100 µg/mL). All cells were maintained in a humidified incubator at 37°C in 5% CO_2_/95% air.

**Figure 2:**
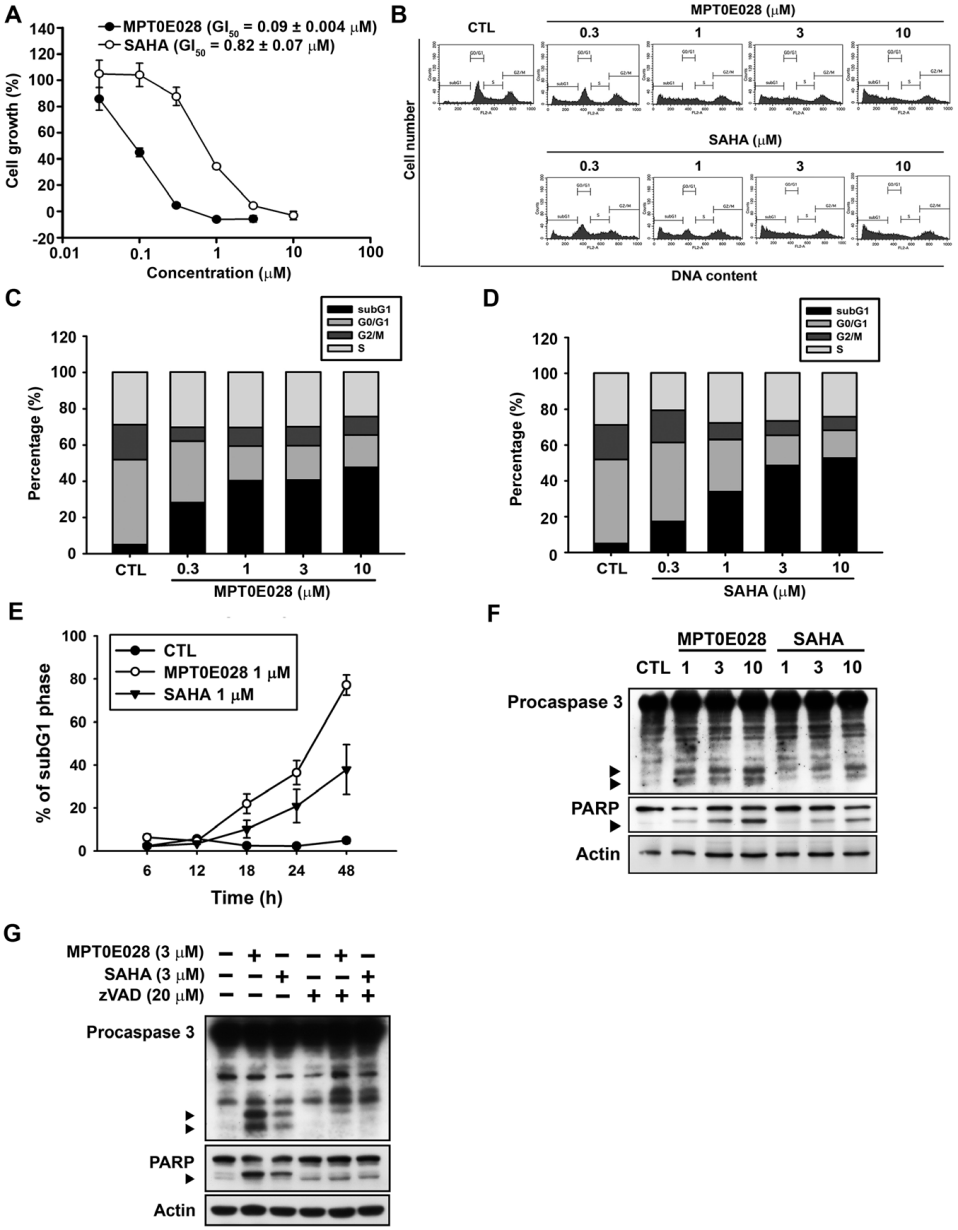
The effects of MPT0E028 on cell growth and cell cycle progression in human HCT116 cells. (A) Concentration-dependent effect of MPT0E028 and SAHA on cell growth. HCT116 cells were incubated without or with the indicated concentrations of MPT0E028 or SAHA for 48 h. Cell growth was evaluated by SRB assay. Data were expressed as mean±S.E.M. of at least 3 independent experiments. (B) Concentration-dependent effects of MPT0E028 and SAHA on cell cycle progression. HCT116 cells were treated without or with the indicated concentrations of MPT0E028 or SAHA for 24 h and were analyzed by flow cytometry for cell cycle distribution. (C, D) Data shown are the means of at least 3 independent experiments. (E) Time-dependent effects of MPT0E028 and SAHA on subG1 population. HCT116 cells were treated without or with 1 µM MPT0E028 or SAHA for the indicated time interval and were analyzed by flow cytometry for subG1 population. (F) MPT0E028 induced-caspase 3 and PARP activation. HCT116 cells were treated without or with the indicated concentration of MPT0E028 or SAHA for 24 h subject to western blot for caspase 3 and PARP analysis. (G) MPT0E028 induced casepase-dependent cell apoptosis. HCT116 cells were treated without or with 3 µM MPTE028, SAHA or 20 µM z-VAD-fmk for 24 h and subjected to western blot for caspase 3 and PARP analysis.

### NCI-60 Cell Lines Screening

The NCI-60 cancer cell lines screening was conducted by the NCI’s Developmental Therapeutics Program (DTP; http://www.dtp.nci.nih.gov/branches/btb/ivclsp.html).

### Sulforhodamine B Assay

HCT116, MDAMB231 and NCI-ADR cells were seeded in 96-well plates in medium with 5% fetal bovine serum overnight. Cells were fixed with 10% trichloroacetic representing cell population at the time of drug treatment (*T*_0_). After incubation with vehicle (0.1% DMSO), MPT0E028 or SAHA for 48 h, cells were fixed with 10% trichloroacetic acid and then stained with sulforhodamine B at 0.4% (w/v) in 1% acetic acid. Excess Sulforhodamine B was washed away by 1% acetic acid and dye-containing cells were lysed with 10 mmol/L Trizma base. The absorbance was read under wavelength of 515 nm. By measuring time zero (*T*_0_), control growth (*C*), and cell growth in the presence of the drug (*T_x_*), the percentage growth was calculated. Percentage growth inhibition was calculated as 100-[(*T_x_*–*T*_0_)/(*C*-*T*_0_)]×100. Growth inhibition of 50% (GI_50_) is determined at the drug concentration that results in 50% reduction of total protein increase in control cells during the compound incubation.

**Table 2:** Activity of MPT0E028 and SAHA against HDACs.

Compound	HDAC1	HDAC2	HDAC8	HDAC4	HDAC6
	Class I			Class IIa	Class IIb
	IC_50_ (nM±SE)				
MPT0E028	53.0±12.0	106.2±23.0	2532.6±314.7	>10000	29.5±6.4
SAHA	102.2±22.1	336.2±91.2	2898.9±128.7	>10000	19.5±3.1

HDAC activity was determined indirectly by measuring the fluorescence generated by a deacetylated fluorogenic peptide product (**Materials and Methods**). Data represent triplicate determinations from three independent experiments (mean±SE). HDAC Class I isoforms: HDAC1, HDAC2, and HDAC8, HDAC Class II isoforms: HDAC4 and HDAC6.

### Crystal Violet Assay

The HUVECs were seeded into 96-well culture plates (5000 cells/well) in quadruplicate. After 24 h incubation, cells were fixed with 0.1% crystal violet/20% methanol, representing cell population at the time of drug treatment (*T*_0_). Other cells were treated with MPT0E028 or SAHA for 48 h. After 48 h incubation at 37°C, cells were stained with 0.1% crystal violet/20% methanol for 10 minutes and washed for 3 times. Then the dye was eluted by 0.1 M sodium citrate/75% ethanol, and the absorbance is measured at 550 nm.

**Figure 3:**
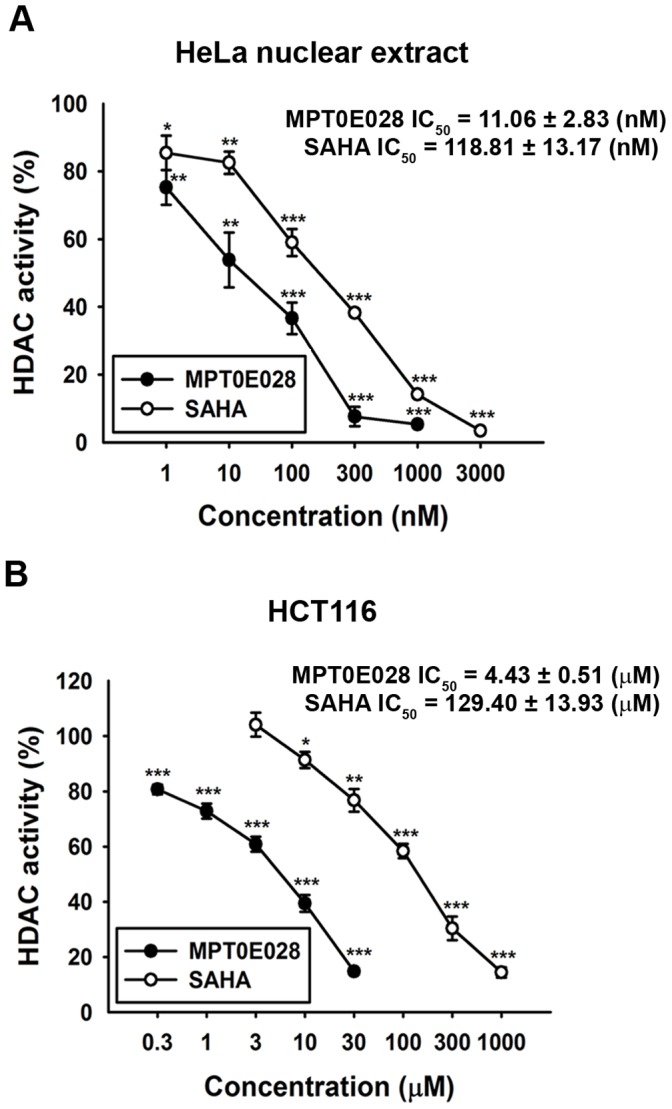
Inhibition of HDACs activity by MPT0E028 and SAHA. (A) Inhibition of HDACs activity in HeLa nuclear extracts. Data were expressed as the mean of at least 3 independent experiments. (B) Inhibition of total HDAC activity by MPT0E028 and SAHA. HCT116 cells were treated with the indicated concentrations of MPT0E028 and SAHA for 24 h, and the nuclear proteins were isolated to determine the inhibition of total HDAC enzyme activity. Data are expressed as the mean±S.E.M. of at least 3 independent experiments.

### FACScan Flow Cytometry

After the treatment of vehicle (0.1% DMSO), MPT0E028 or SAHA for the indicated time courses, the cells were harvested by trypsinization, fixed with 75% (v/v) alcohol at 4°C overnight. After centrifugation, cells were incubated in 0.1 mol/L phosphate-citric acid buffer [0.2 mol/L NaHPO_4_, 0.1 mol/L citric acid (pH 7.8)] for 20 min at room temperature. Then, the cells were centrifuged and resuspended with 0.5 mL PI solution containing Triton X-100 (0.1%, v/v), RNase (100 µg/mL), and PI (80 µg/mL). DNA content was analyzed with the FACScan and CellQuest software (Becton Dickinson).

**Figure 4:**
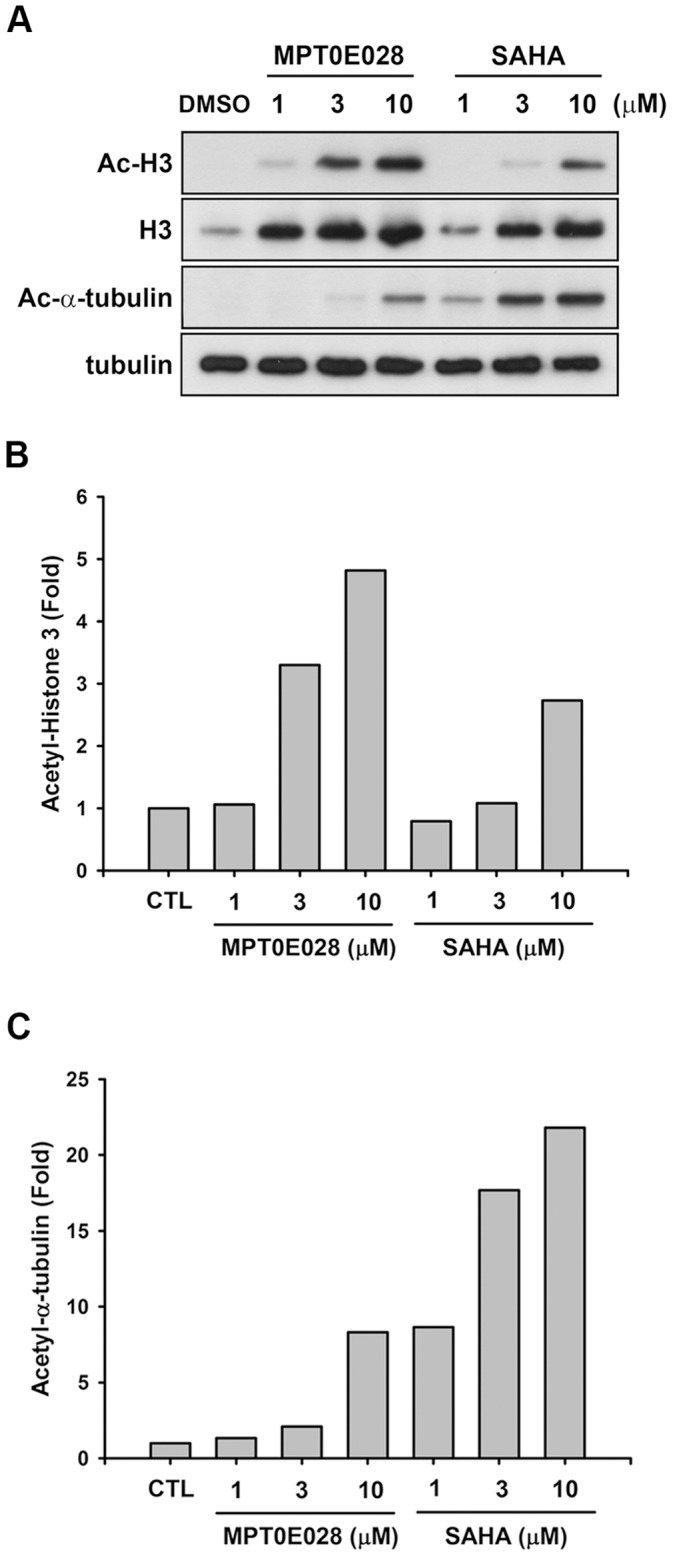
Effect of MPT0E028 and SAHA on α-tubulin and histone H3 acetylation. (A) HCT116 cells were treated with MPT0E028 and SAHA for 24 h at the indicated concentrations. Cell lysates were prepared and subjected to SDS-PAGE and immunoblotting using acetyl-histone H3, histone H3, acetyl-α-tubulin, and α-tubulin antibodies. Quantitative analysis of western blot with ImageQuant (Molecular Dynamics, USA); acetyl-histone H3 (B) and acetyl α-tubulin (C) were analyzed in HCT116 cells.

### HDAC Biochemical Assays

The HDACs *in vitro* activities of recombinant human HDAC 1, 2, 4, 6 and 8 (BPS Biosciences) were detected by fluorigenic release of 7-amino-4-methylcoumarin from substrate upon deacetylase enzymatic activity. Half maximal inhibitory concentration (IC_50_) is determined at the drug concentration that results in 50% reduction of HDAC activity increase in control wells during the compound incubation.

**Figure 5:**
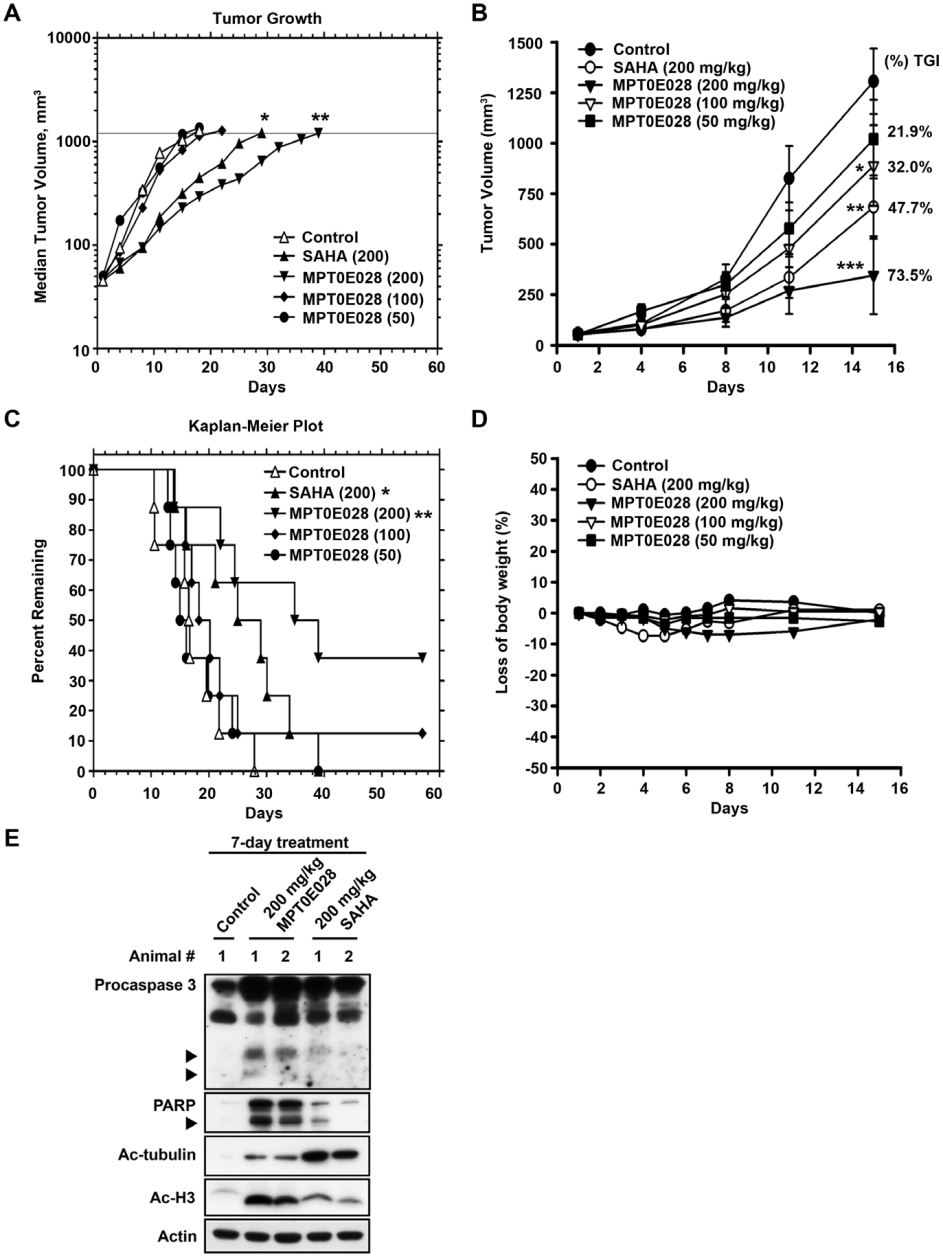
The effect of MPT0E028 on the growth of HCT116 cells *in vivo*. All tumors grew to the 1,200-mm^3^ endpoint volume. (A) Tumors were measured regularly and growth delay was calculated for treatment groups relative to control tumors (TGD). (B) Kaplan-Meier survival analysis was based on the tumor growth endpoint. (C) Inhibition of tumor growth curves represented a mean±SEM and percentage change in mean tumor volume (percent TGI). (D) Body weights were measured daily during the first week and then 2 times per week. The body weight ratio was calculated relative to the baseline measurement. (E) *In vivo* effect of MPT0E028 on the expression of caspase 3, PARP, acetyl-histone H3 and acetyl-µ-tubulin in HCT116 xenograft tumors as determined by western blotting.

### HeLa Nuclear Extract HDAC Activity Assay

The HeLa nuclear extract HDAC activity was measured by using the HDAC Fluorescent Activity Assay Kit (BioVision, CA, USA) according to manufacturer’s instructions [15]. Briefly, the HDAC fluorometric substrate and assay buffer were added to HeLa nuclear extracts in a 96-well format and incubated at 37°C for 30 min. The reaction was stopped by adding lysine developer, and the mixture was incubated for another 30 min at 37°C. Additional negative controls included incubation without the nuclear extract, without the substrate, or without both. TSA at 1 µM served as the positive control. A fluorescence plate reader with excitation at 355 nm and emission at 460 nm was used to quantify HDAC activity. Half maximal inhibitory concentration (IC_50_) is determined at the drug concentration that results in 50% reduction of HeLa HDAC activity increase in control group during the compound incubation.

**Table 3:** Treatment response summary for anti-cancer activity of MPT0E028 and SAHA in the human HCT116 colorectal cancer xenograft model.

Compound (n)	Dose (mg/kg)	Route	Schedule	Median TTE	T-C	%TGD	No. of CR	Logrank Significance	*P* value
Control (8)	–	po	qd to endpoint	16.6	–	–	0		
SAHA (8)	200	po	qd to endpoint	27.0	10	63%	0	*	0.0251
MPT0E028 (8)	200	po	qd to endpoint	36.9	20	122%	3	**	0.0031
MPT0E028 (8)	100	po	qd to endpoint	19.2	2.6	16%	1	ns	0.3084
MPT0E028 (8)	50	po	qd to endpoint	15.5	–	–	0	ns	0.7890

n = number of animals; Study Endpoint = 1200 mm^3^, Days in Progress = 60; TTE = time to endpoint; T-C = difference between median TTE (days) of treated versus control group; %TGD (tumor growth delay) = [(T-C)/C]×100; CR = complete regression; Statistical Significance = Logrank test; ns = not significant; * = *P*<0.05 compare with control. qd = once daily; po = oral administration.

### Total HDACs Enzymatic Activity Assay

Total HDACs enzyme activity was determined using the Boc-Lys(Ac)-AMC fluorometric HDAC activity assay kit (BioVision, Mountain View, CA, USA). HCT116 cells were treated with MPT0E028 and SAHA for 24 h. Cells were then collected and total cell lysates were analyzed using a Fluorometric HDAC Activity Assay Kit (k330-100; BioVision). A fluorescence plate reader with excitation at 355 nm and emission at 460 nm was used to quantify HDAC activity. Half maximal inhibitory concentration (IC_50_) is determined at the drug concentration that results in 50% reduction of total HDAC activity increase in control group.

### Western Blot Analysis

After the treatment of vehicle (0.1% DMSO), MPT0E028 or SAHA at indicated concentrations, cells were washed with chilled PBS. For total lysate, cells were lysed with the 120 µL ice-cold lysis buffer [10 mmol/L Tris-HCl (pH 7.4), 150 mmol/L NaCl, 1 mmol/L EGTA, 1 mmol/L phenylmethylsulfonyl fluoride, 10 µg/mL aprotinin, 10 µg/mL leupeptin, 1 mM sodium orthovandate, 1 mM NaF, and 1% Triton X-100]. Cell lysates were centrifuged at 13,000 rpm for 30 min. For Western blot analysis, the amount of protein (40 µg) was separated by electrophoresis in a 10% or 15% polyacrylamide gel and transferred to a polyvinylidene difluoride (PVDF) membrane. After 1 h incubation at room temperature in PBS/5% nonfat milk, the membrane was washed with PBS/0.1% and incubated with the indicated antibodies at 4°C overnight. After washings with PBS/0.1% Tween 20, the corresponding secondary antibodies were applied to the membranes for 1 h at room temperature. The membranes were then washed with PBS/0.1% Tween 20 and the detection of signal was done with an enhanced chemiluminescence detection kit (Amersham, Buckinghamshire, UK).

### *In vivo* Implantation and Tumor Growth

The human HCT116 colorectal adenocarcinoma cells used for implantation were harvested during log phase growth and resuspended in phosphate-buffered saline at 5×10^7^ cells/mL. Each mouse was injected s.c. in the right flank with 1.0×10^7^ cells (0.2 mL cell suspension). Tumors were monitored twice weekly and then daily as their volumes approached 1,200 mm^3^. Tumor size, in mm^3^, was calculated from: where *w = *width and *l = *length in mm of the tumor. Tumor volume = (*w^2^*×*l*)/2. All animal experiments followed ethical standards, and protocols have been reviewed and approved by Animal Use and Management Committee of National Taiwan University (IACUC Approval No: 20100225).

### Mice

Female nude-athymic mice were 8 weeks old, and had a body weight (BW) range of 17.2–22.0 g, on D1 of the study. The animals were fed ad libitum water (reverse osmosis, 1 ppm Cl) and PicoLab Rodent Diet 20 Modified and Irradiated Lab Diet® consisting of 20.0% crude protein, 9.9% crude fat, and 4.7% crude fiber. The mice were housed on National Taiwan University Laboratory Animal Center, NTUMC, on a 12-hour light cycle at 21–23°C and 60–85% humidity.

### Statistical and Graphical Analyses

The logrank test was used to determine the statistical significance of the difference between the TTE values of two groups, except any NTR deaths. Statistical and graphical analyses were performed with Prism 3.03 (GraphPad) for Windows. The two-tailed statistical analyses were conducted at *P* = 0.05. Kaplan-Meier plots show the percentage of animals remaining in the study versus time. The Kaplan-Meier plots use the same data set as the logrank test.

The tumor growth curves show the group median tumor volume, on a log scale as a function of time. When an animal exits the study due to tumor size or TR death, the final tumor volume recorded for the animal is included with the data used to calculate the median at subsequent time points. Therefore, the final median tumor volume shown by the curve may differ from the MTV, which is the median tumor volume for mice remaining in the study on the last day (excluding all with tumors that have attained the endpoint). If more than one TR death occurs in a group, the tumor growth curves are truncated at the time of the last measurement that precedes the second TR death. Tumor growth curves are also truncated when the tumors in more than 50% of the assessable animals in a group have grown to the endpoint volume. (*p<0.05; **p<0.01; ***p<0.001; compared with the respective control group).

### *In vitro* Statistical Analysis

Data were expressed as mean±SEM of the indicated number for separate experiments. Statistical analysis of data was performed with one-way ANOVA followed by the *t* test, and *p*<0.05 were considered significant. (*p<0.05; **p<0.01; ***p<0.001; compared with the respective control group).

## Results

### The Synthesis of MPT0E028

The synthesis of MPT0E028 (**1**) is depicted in Figure 1. Commercially available methyl indole-5-carboxylate (**2**) was reacted with sodium cyanoborohydride in the presence of acetic acid afforded the methyl indoline-5-carboxylate (**3**) in 93% yield. The *N*1-sulfonylation of indoline (**3**) with benzenesulfonyl chloride in pyridine and then LiAlH_4_-mediated C5-ester reduction, followed by the PDC oxidation produced the desired 1-benzenesulfonylindoline-5-carbaldehyde (**4**) in 42% yield (3 steps). Wittig reaction of methyl(triphenylphosphoranylidene)acetate with the indoline-5-carboxyaldehyde **4**T and treatment of lithium hydroxide (LiOH) in MeOH gave cinnamic acid (**5**) in 73% yield. The conversion of carboxylic acid (**5**) to the hydroxamic acid (**1**) was accomplished in 72% yield by the treatment of NH_2_OTHP in the presence of PYBOP and Et_3_N, followed by trifluoroacetic acid-mediated deprotection. The detailed experimental information for the preparation of MPT0E028 is available in the supplemental section.

### The Effect of MPT0E028 on Proliferation of Cancer Cell Lines

To determine whether MPT0E028 exhibits anti-cancer activity in human cancer cells, we tested the anti-proliferative activity of MTP0E028 using the NCI-60 cancer cell lines screening panel [16]. As shown in Table 1, MPT0E028 significantly inhibited cell proliferation in all human cancer cell lines examined at micromolar concentrations, but was especially potent in HCT116 cell line. Therefore, we chose HCT116 cells for further studies of the anti-cancer activity of MPT0E028 *in vitro* and *in vivo*.

### MPT0E028 Significantly Induced HCT116 Cell Apoptosis

First, we demonstrated that MPT0E028 inhibits the growth of HCT116 cells in a concentration-dependent manner using SRB assay (GI_50_ = 0.09±0.004 µM) (Figure 2A). Two other cell lines, MDA-MB-231 and NCI-ADR from the NCI-60 screening panel, were also chosen to analyze the sensitivity of MPT0E028 (MDAMB231, GI_50_ = 0.19±0.04 µM; NCI-ADR, GI_50_ = 0.14±0.02 µM). MPT0E028 also inhibited cell proliferation significantly in these two cell lines (Data S2A, S2B) but showed less sensitive compared to HCT116. We also determined the safety margin of MPT0E028 in human normal cells (e.g. HUVEC) and found that MPT0E028 show 20–40 folds less sensitive against normal cells (GI_50_ = 3.57±0.45 µM), suggesting MPT0E028 specifically targets malignant tumor cells (Data S2C). To investigate the effect of MPT0E028 on cell cycle progression, cellular DNA content was measured by flow cytometry. We showed that treatment with MPT0E028 increased the number of cells in the sub-G1 phase of the cell cycle (Figure 2B and 2C). The reference compound was an HDACi, suberoylanilide hydroxamic acid (SAHA; vorinostat), with a GI_50_ of 0.82±0.07 µM, which exhibited antiproliferative activity (Figure 2A) and induced apoptosis in HCT116 cells (Figure 2B and 2D) in a concentration-dependent manner but showed less effective compared to MPT0E028. As shown in Figure 2E, MPT0E028 also induced sub-G1 population in a time-dependent manner, while SAHA showed weaker effect and delayed cytotoxicity. MPT0E028 also induced caspase 3 and PARP activation in a concentration-dependent manner (Figure 2F). MPT0E028-induced apoptosis was abolished when cotreatment with caspase-inhibitor z-VAD-fmk, suggesting that MPT0E028 induced apoptosis through caspase-dependent pathway. These results suggested that MPT0E028 induced HCT116 cells cytotoxicity through the apoptosis pathway.

### MPT0E028 is an Inhibitor of HDACs Activity

We next examined the effect of MPT0E028 on the activity of HDACs, using a panel of human recombinant HDAC proteins, and compared its activity to SAHA. As shown in Table 2, MPT0E028 significantly inhibited HDAC1 and HDAC2 from class I as well as HDAC6 from class IIb at low nanomolar concentrations. While MPT0E028 was more potent than SAHA against recombinant HDAC1 and 2, it has little activity against recombinant HDAC4 and HDAC8, which is similar to the inhibitory pattern of SAHA. We confirmed that MPT0E028 inhibited nuclear HDAC activity in the cervical adenocarcinoma cell line HeLa (IC_50_ = 11.1±2.8 nM) and in HCT116 cells (IC_50_ = 4.43±0.5 µM), which were approximately 9–30 times more potent than SAHA (IC_50_ = 118.8±13.2 nM and 129.4±13.9 µM, respectively) (Figure 3A and 3B). The potent HDACs inhibitory effect of MPT0E028 could also be observed in MDAMB231 and NCI-ADR cells (Data S3). These results suggested that MPT0E028 is a novel and potent HDAC inhibitor.

### Effects of MPT0E028 on α-tubulin and Histone H3 Acetylation in HCT116 Cells

Because α-tubulin and histone H3 are common downstream targets of HDACs, we examined the effects of MPT0E028 on α-tubulin and histone H3 acetylation in HCT116 cells using western blot analysis. As shown in Figure 4, MPT0E028 induced stronger hyperacetylation of histone H3 than that of SAHA (Figure 4A and 4B), which is consistent with its potent inhibitory effect on class I HDAC1 and 2. In contrast, SAHA exhibited more potent α-tubulin acetylation than MPT0E028 (Figure 4A and 4C), suggesting that SAHA is more potent against class IIb HDAC6, which is consistent with our finding in Table 2.

### MPT0E028 Inhibits Growth of Human Colon Cancer Xenografts *in vivo*

Finally, we evaluated the efficiency of MPT0E028 and SAHA using *in vivo* tumor HCT116 xenografts in a nude mice model (Figure 5A and 5B) (Table 3). Once a tumor was palpable with the size of approximately 55 mm^3^, mice were randomized into vehicle control and treatment groups (8 mice per group). Control mice received the vehicle (0.5% carboxymethyl cellulose +0.1% Tween 80). Tumors in all 3 groups were allowed to reach an endpoint volume of 1,200 mm^3^. The median time to endpoint (TTE) for control mice was 16.6 days. In mice that were orally treated with SAHA (200 mg/kg, daily), the TTE increased to 27.0 days, which corresponds to a T-C of 10.0 days and a percent tumor growth delay (TGD) of 63%. According to the logrank analysis, SAHA produced significant antitumor activity (*P* = 0.0251). Oral treatment with MPT0E028 (200, 100, and 50 mg/kg, daily) increased the median TTE to 36.9, 19.2, and 15.5 days, respectively, corresponding to a T-C of 20, 2.6, and 0 days and a percent TGD of 122%, 16%, and 0%, respectively. Based on the logrank analysis, MPT0E028 exhibits significant antitumor activity at 200 mg/kg (*P* = 0.0031). Notably, 3 mice in a group that were treated with 200 mg/kg MPT0E028 and 1 mouse in another group that was treated with 100 mg/kg MPT0E028 showed complete tumor regression after treatment (Table 3). In addition, mice were also dosed once a day for 15 days via oral administration to evaluate tumor growth inhibition after MPT0E028 treatment. we found that the growth of HCT116 cancer cells xenografts was suppressed by 73.5%, 32.0%, and 21.9% (percent tumor growth inhibition [TGI] values) after treatment with MPT0E028 at 200, 100, and 50 mg/kg, respectively, whereas SAHA at 200 mg/kg suppressed tumor growth by 47.7% (%TGI) (Figure 5C). No significant difference in body weight or other adverse effects were observed upon treatment with MPT0E028 (Figure 5D). We also determined the effect of MPT0E028 on the expression of caspase3, PARP, acetyl-histone H3 and acetyl-µ-tubulin *in vivo* in HCT116 xenograft tumor tissues. Mice were treated with 200 mg/kg MPT0E028 or SAHA for 7 days (oral, daily) and then tumors were carefully removed and subjected to western blot analysis. As shown in Figure 5E, caspase 3, PARP and acetyl-histone H3 were substantially induced in MPT0E028-treated group while acetyl-µ-tubulin were found more profoundly in SAHA-treated group, which is consistent with our finding *in vitro*. Taken together, MPT0E028 induced a dose-dependent delay and inhibition of tumor growth and displayed stronger anti-tumor activity *in vivo* than SAHA.

## Discussion

HDACs are important targets for cancer therapy because of their ability to transcriptionally regulate the expression of genes that are involved in cell proliferation, differentiation, and apoptosis [17], [18]. HDACi are currently in clinical trials for the treatment of solid tumors and in leukemia patients. Two HDACi, SAHA and FK228, have received the US FDA approval for the treatment of patients with cutaneous T-cell lymphoma. In this study, we report the synthesis of a new chemical compound 3-(1-benzenesulfonyl-2,3-dihydro-1H-indol-5-yl)-*N*-hydroxy-acrylamide (MPT0E028), that has a potent HDACi activity. MPT0E028 was designed based on our prior research with microtubule destabilizing agents using indoline-1-arylsulfonamides as a core structure [13], [14]. After coupling an *N*-hydroxyacrylamide functional group at the 5-position of indoline ring, we afforded the desired MPT0E028.

Furthermore, we evaluated the anti-cancer activity of MPT0E028 i*n vitro* and *in vivo*. We found that MPT0E028 induced cytotoxicity in numerous human cancer cell lines from the NCI-60 panels and performed mechanistic studies in HCT116 cells, which showed high sensitivity to MPT0E028. When compared to SAHA, treatment with MPT0E028 induced stronger inhibition of cancer cell (GI_50_ = 0.82±0.07 µM and 0.09±0.004 µM, respectively) but not normal cell growth (GI_50_ = 3.57±0.45 µM) and produced a significantly higher number of sub-G1 (apoptotic) cells as determined by flow cytometry. Also, MPT0E028 induced more profoundly caspase 3 and PARP activation. Taken together, MPT0E028 inhibits cancer cells growth and induces apoptotic cell-death.

We demonstrated that MPT0E028 inhibits the activity of HDAC1, HDAC2 and HDAC8 in class I as well as HDAC6 in class IIb, but not HDAC4 in class IIa, and consistently induces acetylation of histone H3 and α-tubulin. Class I HDACs have been shown to be overexpressed in human colorectal cancer cells [19], which may contribute to perturbed cancer cell proliferation, differentiation, and apoptosis by both epigenetic or non-epigenetic modification [20], [21]. Inhibition of HDAC activity induced the intrinsic and extrinsic apoptotic pathway, leading to cancer cell death [22], [23]. In addition, HDACi could induce expression of p21 by stabilizing and inducing transcriptional activity of RUNX3, leading to induction of cancer cell apoptosis [24], [25]. The inhibition of HDAC activity by MPT0E028 was 10–30 times stronger than that by SAHA in HeLa and HCT116 cells.

Finally, we examined the efficiency of MPT0E028 against the human HCT116 colorectal adenocarcinoma cell growth in mice. Median tumor growth and Kaplan-Meier curve analysis demonstrated strong antitumor activity of MPT0E028. Daily administration of MPT0E028 resulted in significant antitumor activity. Moreover, compared to SAHA, MPT0E028 displayed stronger anti-tumor activity. Based on these results, MPT0E028 is a novel synthetic HDACi with unique pharmacologic properties that should be tested in human colorectal cancer therapy.

In summary, we have identified a novel inhibitor of HDAC activity, MPT0E028, with antitumor activity *in vitro* and *in vivo*. The results presented here show that MPT0E028 inhibits HDACs activity and has antitumor properties that are more potent than SAHA, which is currently in clinical use in subcutaneous T-cell lymphoma. Thus, MPT0E028 is suitable for further testing as a novel anti-cancer agent.

## Supporting Information

Data S1 **Chemistry.** Nuclear magnetic resonance (^1^H NMR) spectra were obtained with Bruker DRX-500 spectrometer (operating at 500 MHz), with chemical shift in parts per million (ppm, *δ*) downfield from TMS as an internal standard. High-resolution mass spectra (HRMS) were measured with a JEOL (JMS-700) electron impact (EI) mass spectrometer. Flash column chromatography was done using silica gel (Merck Kieselgel 60, No. 9385, 230–400 mesh ASTM). All reactions were carried out under an atmosphere of dry nitrogen. **2,3-Dihydro-1H-indole-5-carboxylic acid methyl ester** **(3):** To a stirred solution of methyl indole-5-carboxylate (**2**) (0.30 g, 1.71 mmol) in AcOH (2 mL), sodium cyanoborohydride (0.16 g, 2.57 mmol) was added to the reaction mixture at 0°C. The reaction was warmed to room temperature and stirred for 2 h. The reaction was quenched with water at 0°C, concentrated NaOH was added up to pH 10. The aqueous layer was extracted with CH_2_Cl_2_ (15 mL×3). The combined organic layer was dried over anhydrous MgSO_4_ and concentrated under reduced pressure to give a yellow residue, which was purified by silica gel chromatography (EtOAc: *n*-hexane = 1∶2) to afford **3** (0.28 g), yield 93%. ^1^H NMR (500 MHz, CDCl_3_): δ 3.06 (t, *J* = 8.5 Hz, 2H), 3.65 (t, *J* = 8.5 Hz, 2H), 3.84 (s, 3H), 6.53–6.55 (m, 1H), 7.75–7.76 (m, 2H). **1-Benzenesulfonyl-2,3-dihydro-1H-indole-5-carbaldehyde** **(4)**: To a solution of **3** (0.28 g, 1.58 mmol) in pyridine (2 mL), benzenesulfonyl chloride (0.40 ml, 3.16 mmol) was added. The reaction mixture was refluxed overnight. The mixture was then purified by silica gel chromatography (EtOAc: *n*-hexane = 1∶3) to afford the 1-benzenesulfonylindoline (0.40 g).^ 1^H NMR (500 MHz, CDCl_3_): δ 2.99 (t, *J* = 8.6 Hz, 2H), 3.87 (s, 3H), 3.97 (t, *J* = 8.6 Hz, 2H), 7.45–7.48 (m, 2H), 7.56–7.59 (m, 1H), 7.66 (d, *J* = 8.5 Hz, 1H), 7.75 (s, 1H), 7.82 (d, *J* = 7.7 Hz, 2H), 7.90 (d, *J* = 7.9 Hz, 1H). LAH (0.10 g, 2.52 mmol) was added to a solution of 1-benzenesulfonylindoline (0.40 g, 1.26 mmol) in THF (10 mL) at 0°C. The reaction mixture was warmed to room temperature and stirred for 2 h before it was quenched with water and then extracted with CH_2_Cl_2_ (15 mL×3). The combined organic layer was dried over anhydrous MgSO_4_ and concentrated under reduced pressure. The residue was further treated with PDC (0.63 g, 1.66 mmol)-mediated oxidation in the presence of molecular sieves (0.63 g) and CH_2_Cl_2_ (10 mL) stirring at room temperature overnight. The reaction mixture was filtered through celite and purified by silica gel chromatography (EtOAc: *n*-hexane = 1∶2) to afford **4** (0.19 g), 42% yield. ^1^H NMR (500 MHz, CDCl_3_): δ 3.05 (t, *J* = 8.6 Hz, 2H), 4.01 (t, *J* = 8.7 Hz, 2H), 7.46–7,49 (m, 2H), 7.58–7.62 (m, 2H), 7.71 (d, *J* = 8.3 Hz, 1H), 7.75 (d, *J* = 8.3 Hz, 1H), 7.84 (d, *J* = 7.8 Hz, 2H), 9.85 (s, 1H). **3-(1-Benzenesulfonyl-2,3-dihydro-1H-indol-5-yl)-acrylic acid** **(5)**: To a solution of **4** (0.19 g, 0.66 mmol) in CH_2_Cl_2_ (10 mL) was treated with methyl (triphenylphosphoranylidene) acetate (0.27 g, 0.79 mmol). The reaction mixture was stirred at room temperature for 3 h, was then quenched with water, and extracted by CH_2_Cl_2_ (15 mL×3). The combined organic layer was dried over anhydrous MgSO_4_ and concentrated under reduced pressure to give a yellow residue, which was then purified by silica gel chromatography (EtOAc: *n*-hexane = 1∶3) to afford the acrylic acid methyl ester (0.20 g). 1 M LiOH aqueous solution (1.16 ml, 1.16 mmol) was added to a solution of acrylic acid methyl ester (0.20 g, 0.58 mmol) in dioxane (15 mL). The reaction mixture was stirred at 40°C overnight before it was concentrated under reduced pressure. The residue was dissolved in water and concentrated HCl was added up to acidic pH to give the precipitation, which was dried by vacuum to afford **5** (0.16 g), 73% yield. ^1^H NMR (500 MHz, CD_3_OD): δ 2.92 (t, *J* = 8.5 Hz, 2H), 3.96 (t, *J* = 8.5 Hz, 2H), 6.33 (d, *J* = 15.9 Hz, 1H), 7.38 (s, 1H), 7.41 (d, *J* = 8.5 Hz, 1H), 7.50–7.53 (m, 2H), 7.55 (d, *J* = 16.1 Hz, 1H), 7.58–7.64 (m, 2H), 7.82 (d, *J* = 7.6 Hz, 2H). **3-(1-Benzenesulfonyl-2,3-dihydro-1H-indol-5-yl)-N-hydroxy-acrylamide** (**MPT0E028, 1)**: NH_2_OTHP (0.05 g, 0.44 mmol) was added to a stirred solution of **5** (0.12 g, 0.37 mmol), PYBOP (0.20 g, 0.39 mmol), triethylamine (0.12 ml, 0.88 mmol) in DMF (1.5 mL). The reaction mixture was stirred at room temperature for 1 h before it was quenched with water, followed by extraction with EtOAc (15 mL×3). The combined organic layer was dried over anhydrous MgSO_4_ and concentrated under reduced pressure. The residue was purified by silica gel chromatography (CH_2_Cl_2_: CH_3_OH = 30∶1 : 1%NH_3(aq)_) to give a white solid, which was treated with TFA (1.13 ml, 15.21 mmol) in the presence of CH_3_OH (25 mL) and stirred overnight at room temperature. The reaction mixture was concentrated under reduced pressure to give a white residue, which was recrystallized by EtOAc/CH_3_OH to afford compound **1** (0.09 g), 72% yield. mp: 162–164°C. ^1^H NMR (500 MHz, CD_3_OD): δ 2.91 (t, *J* = 8.5 Hz, 2H), 3.96 (t, *J* = 8.4 Hz, 2H), 6.32 (d, *J* = 15.8 Hz, 1H), 7.32 (s, 1H), 7.37–7.39 (m, 1H), 7.46 (d, *J* = 15.7 Hz, 1H), 7.50–7.53 (m, 2H), 7.58–7.64 (m, 2H), 7.82 (d, *J* = 7.8 Hz, 2H). MS (EI) *m*/*z*: 170 (100%), 344 (M^+^, 3.21%). HRMS (EI) for C_17_H_16_N_2_O_4_S (M^+^): calcd, 344.0831; found, 344.0829.
(PDF)

Data S2 **The effects of MPT0E028 on cell growth in human MDAMB231, NCI-ADR and HUVEC cells.** Concentration-dependent effect of MPT0E028 and SAHA on cell growth in (A) MDAMB231, (B) NCI-ADR and (C) HUVEC cells. Cells were incubated without or with the indicated concentrations of MPT0E028 or SAHA for 48 h. Cell growth was evaluated by SRB and crystal violet assay. Data were expressed as mean±S.E.M. of at least 3 independent experiments.
(PDF)

Data S3 **Inhibition of total HDAC activity by MPT0E028 and SAHA in MDAMB231 and NCI-ADR cells.** (A) MDAMB231 and (B) NCI-ADR cells were treated with the indicated concentrations of MPT0E028 and SAHA for 24 h, and the total lysate were subjected to total HDAC enzyme activity detection. Data are expressed as the mean±S.E.M. of at least 3 independent experiments.
(PDF)
