# Correction: Anticancer Activity of MPT0E028, a Novel Potent Histone Deacetylase Inhibitor, in Human Colorectal Cancer HCT116 Cells *In Vitro* and *In Vivo*

**DOI:** 10.1371/annotation/ab4fff87-6a32-4718-aa4c-91658f164b8d

**Published:** 2012-09-19

**Authors:** Han-Li Huang, Hsueh-Yun Lee, An-Chi Tsai, Chieh-Yu Peng, Mei-Jung Lai, Jing-Chi Wang, Shiow-Lin Pan, Che-Ming Teng, Jing-Ping Liou

The first author's name was spelled incorrectly. The correct name is: Han-Li Huang. 

