# Supplementary material for: Anticancer Activity of MPT0E028, a Novel Potent Histone Deacetylase Inhibitor, in Human Colorectal Cancer HCT116 Cells In Vitro and In Vivo
Source: PLoS One. 2012 Aug 22;7(8):e43645. doi: 10.1371/journal.pone.0043645 (PMC3425516; doi:10.1371/journal.pone.0043645)
Supplement: Data S1 — Chemistry. Nuclear magnetic resonance (1H NMR) spectra were obtained with Bruker DRX-500 spectrometer (operating at 500 MHz), with chemical shift in parts per million (ppm, δ) downfield from TMS as an internal standard. High-resolution mass spectra (HRMS) were measured with a JEOL (JMS-700) electron impact (EI) mass spectrometer. Flash column chromatography was done using silica gel (Merck Kieselgel 60, No. 9385, 230–400 mesh ASTM). All reactions were carried out under an atmosphere of dry nitrogen. 2,3-Dihydro-1H-indole-5-carboxylic acid methyl ester (3): To a stirred solution of methyl indole-5-carboxylate (2) (0.30 g, 1.71 mmol) in AcOH (2 mL), sodium cyanoborohydride (0.16 g, 2.57 mmol) was added to the reaction mixture at 0°C. The reaction was warmed to room temperature and stirred for 2 h. The reaction was quenched with water at 0°C, concentrated NaOH was added up to pH 10. The aqueous layer was extracted with CH2Cl2 (15 mL×3). The combined organic layer was dried over anhydrous MgSO4 and concentrated under reduced pressure to give a yellow residue, which was purified by silica gel chromatography (EtOAc: n-hexane = 1∶2) to afford 3 (0.28 g), yield 93%. 1H NMR (500 MHz, CDCl3): δ 3.06 (t, J = 8.5 Hz, 2H), 3.65 (t, J = 8.5 Hz, 2H), 3.84 (s, 3H), 6.53–6.55 (m, 1H), 7.75–7.76 (m, 2H). 1-Benzenesulfonyl-2,3-dihydro-1H-indole-5-carbaldehyde (4): To a solution of 3 (0.28 g, 1.58 mmol) in pyridine (2 mL), benzenesulfonyl chloride (0.40 ml, 3.16 mmol) was added. The reaction mixture was refluxed overnight. The mixture was then purified by silica gel chromatography (EtOAc: n-hexane = 1∶3) to afford the 1-benzenesulfonylindoline (0.40 g). 1H NMR (500 MHz, CDCl3): δ 2.99 (t, J = 8.6 Hz, 2H), 3.87 (s, 3H), 3.97 (t, J = 8.6 Hz, 2H), 7.45–7.48 (m, 2H), 7.56–7.59 (m, 1H), 7.66 (d, J = 8.5 Hz, 1H), 7.75 (s, 1H), 7.82 (d, J = 7.7 Hz, 2H), 7.90 (d, J = 7.9 Hz, 1H). LAH (0.10 g, 2.52 mmol) was added to a solution of 1-benzenesulfonylindoline (0.40 g, 1.26 mmol) in THF (10 mL) at [file pone.0043645.s001.pdf]

Supplemental data:

Chemistry

Nuclear magnetic resonance (^1H NMR) spectra were obtained with Bruker DRX-500 spectrometer (operating at 500 MHz), with chemical shift in parts per million (ppm, δ) downfield from TMS as an internal standard. High-resolution mass spectra (HRMS) were measured with a JEOL (JMS-700) electron impact (EI) mass spectrometer. Flash column chromatography was done using silica gel (Merck Kieselgel 60, No. 9385, 230-400 mesh ASTM). All reactions were carried out under an atmosphere of dry nitrogen.

2,3-Dihydro-1H-indole-5-carboxylic acid methyl ester (3): To a stirred solution of methyl indole-5-carboxylate (**2**) (0.30 g, 1.71 mmol) in AcOH (2 mL), sodium cyanoborohydride (0.16 g, 2.57 mmol) was added to the reaction mixture at 0°C. The reaction was warmed to room temperature and stirred for 2 h. The reaction was quenched with water at 0°C, concentrated NaOH was added up to pH 10. The aqueous layer was extracted with CH_2Cl_2 (15 mL × 3). The combined organic layer was dried over anhydrous $MgSO_4$ and concentrated under reduced pressure to give a yellow residue, which was purified by silica gel chromatography (EtOAc: *n*-hexane =

1 : 2) to afford **3** (0.28 g), yield 93%. ^{1}H NMR (500MHz, $CDCl_3$): δ 3.06 (t, *J* = 8.5 Hz, 2H), 3.65 (t, *J* = 8.5 Hz, 2H), 3.84 (s, 3H), 6.53-6.55 (m, 1H), 7.75-7.76 (m, 2H).

1-Benzenesulfonyl-2,3-dihydro-1H-indole-5-carbaldehyde (4): To a solution of **3** (0.28 g, 1.58 mmol) in pyridine (2 mL), benzenesulfonyl chloride (0.40 ml, 3.16 mmol) was added. The reaction mixture was refluxed overnight. The mixture was then purified by silica gel chromatography (EtOAc: *n*-hexane = 1 : 3) to afford the 1-benzenesulfonylindoline (0.40 g). ^{1}H NMR (500MHz, $CDCl_3$): δ 2.99 (t, *J* = 8.6 Hz, 2H), 3.87 (s, 3H), 3.97 (t, *J* = 8.6 Hz, 2H), 7.45-7.48 (m, 2H), 7.56-7.59 (m, 1H), 7.66 (d, *J* = 8.5 Hz, 1H), 7.75 (s, 1H), 7.82 (d, *J* = 7.7 Hz, 2H), 7.90 (d, *J* = 7.9 Hz, 1H). LAH (0.10 g, 2.52 mmol) was added to a solution of 1-benzenesulfonylindoline (0.40 g, 1.26 mmol) in THF (10 mL) at 0 °C. The reaction mixture was warmed to room temperature and stirred for 2 h before it was quenched with water and then extracted with CH_2Cl_2 (15 mL × 3). The combined organic layer was dried over anhydrous $MgSO_4$ and concentrated under reduced pressure. The residue was further treated with PDC (0.63 g, 1.66 mmol)-mediated oxidation in the presence of molecular sieves (0.63g) and CH_2Cl_2 (10 mL) stirring at room temperature overnight. The reaction mixture was filtered through celite and purified by silica gel chromatography (EtOAc: *n*-hexane = 1 : 2) to afford **4** (0.19 g), 42% yield. ^{1}H NMR (500MHz, $CDCl_3$): δ 3.05

(t, *J* = 8.6 Hz, 2H), 4.01 (t, *J* = 8.7 Hz, 2H), 7.46-7,49 (m, 2H), 7.58-7.62 (m, 2H), 7.71 (d, *J* = 8.3 Hz, 1H), 7.75 (d, *J* = 8.3 Hz, 1H), 7.84 (d, *J* = 7.8 Hz, 2H), 9.85 (s, 1H).

3-(1-Benzenesulfonyl-2,3-dihydro-1H-indol-5-yl)-acrylic acid (5): To a solution of **4** (0.19g, 0.66 mmol) in CH_2Cl_2 (10 mL) was treated with methyl (triphenylphosphoranylidene) acetate (0.27 g, 0.79 mmol). The reaction mixture was stirred at room temperature for 3h, was then quenched with water, and extracted by CH_2Cl_2 (15 mL × 3). The combined organic layer was dried over anhydrous $MgSO_4$ and concentrated under reduced pressure to give a yellow residue, which was then purified by silica gel chromatography (EtOAc: *n*-hexane = 1 : 3) to afford the acrylic acid methyl ester (0.20 g). 1M LiOH aqueous solution (1.16 ml, 1.16 mmol) was added to a solution of acrylic acid methyl ester (0.20g, 0.58 mmol) in dioxane (15 mL). The reaction mixture was stirred at 40 °C overnight before it was concentrated under reduced pressure. The residue was dissolved in water and concentrated HCl was added up to acidic pH to give the precipitation, which was dried by vacuum to afford **5** (0.16 g), 73% yield. ^{1}H NMR (500MHz, CD_3OD): δ 2.92 (t, *J* = 8.5 Hz, 2H), 3.96 (t, *J* = 8.5 Hz, 2H), 6.33 (d, *J* = 15.9 Hz, 1H), 7.38 (s, 1H), 7.41 (d, *J* = 8.5 Hz, 1H),

7.50-7.53 (m, 2H), 7.55 (d, *J* = 16.1 Hz, 1H), 7.58-7.64 (m, 2H), 7.82 (d, *J* = 7.6 Hz, 2H).

3-(1-Benzenesulfonyl-2,3-dihydro-1H-indol-5-yl)-N-hydroxy-acrylamide (MPT0E028, 1): NH_2OTHP (0.05 g, 0.44 mmol) was added to a stirred solution of **5** (0.12 g, 0.37 mmol), PYBOP (0.20 g, 0.39 mmol), triethylamine (0.12 ml, 0.88 mmol) in DMF (1.5 mL). The reaction mixture was stirred at room temperature for 1 h before it was quenched with water, followed by extraction with EtOAc (15 mL × 3). The combined organic layer was dried over anhydrous $MgSO_4$ and concentrated under reduced pressure. The residue was purified by silica gel chromatography (CH_2Cl_2: CH_3OH = 30 : 1 : 1%$NH_{3(aq)}$) to give a white solid, which was treated with TFA (1.13 ml, 15.21 mmol) in the presence of CH_3OH (25 mL) and stirred overnight at room temperature. The reaction mixture was concentrated under reduced pressure to give a white residue, which was recrystallized by EtOAc/CH_3OH to afford compound **1** (0.09 g), 72% yield. mp: 162-164 °C. ^1H NMR (500MHz, CD_3OD): δ 2.91 (t, *J* = 8.5 Hz, 2H), 3.96 (t, *J* = 8.4 Hz, 2H), 6.32 (d, *J* = 15.8 Hz, 1H), 7.32 (s, 1H), 7.37-7.39 (m, 1H), 7.46 (d, *J* = 15.7 Hz, 1H), 7.50-7.53 (m, 2H), 7.58-7.64 (m, 2H), 7.82 (d, *J* = 7.8 Hz, 2H). MS (EI) *m*/*z*: 170 (100%), 344 (M^+, 3.21%). HRMS (EI) for $C_{17}H_{16}N_2O_4S$ (M^+): calcd, 344.0831; found, 344.0829.
