# Supplementary material for: Anticancer Activity of MPT0E028, a Novel Potent Histone Deacetylase Inhibitor, in Human Colorectal Cancer HCT116 Cells In Vitro and In Vivo
Source: PLoS One. 2012 Aug 22;7(8):e43645. doi: 10.1371/journal.pone.0043645 (PMC3425516; doi:10.1371/journal.pone.0043645)
Supplement: Data S2 — The effects of MPT0E028 on cell growth in human MDAMB231, NCI-ADR and HUVEC cells. Concentration-dependent effect of MPT0E028 and SAHA on cell growth in (A) MDAMB231, (B) NCI-ADR and (C) HUVEC cells. Cells were incubated without or with the indicated concentrations of MPT0E028 or SAHA for 48 h. Cell growth was evaluated by SRB and crystal violet assay. Data were expressed as mean±S.E.M. of at least 3 independent experiments. (PDF) [file pone.0043645.s002.pdf]

Supplemental data 2

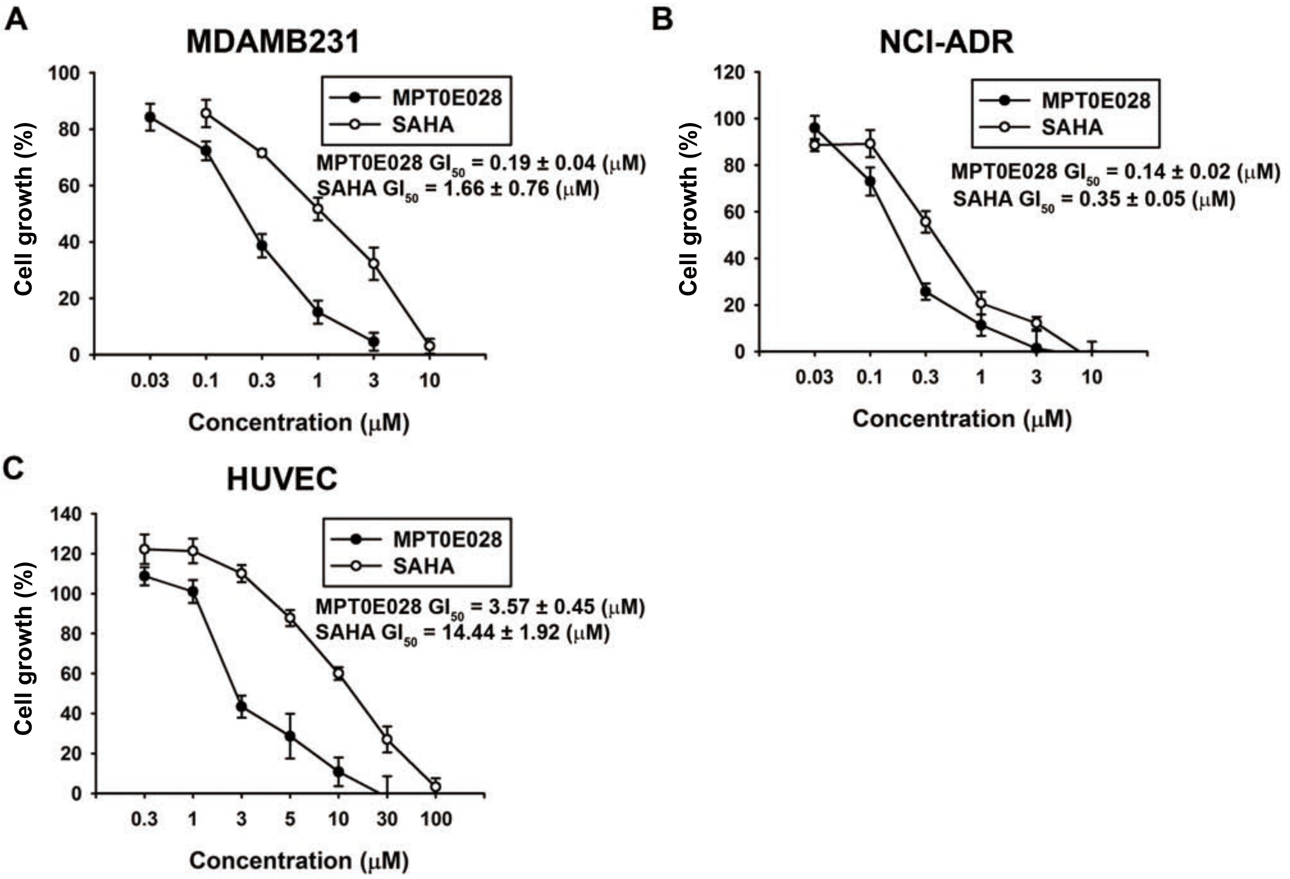

Supplemental data 2. The effects of MPT0E028 on cell growth in human MDAMB231, NCI-ADR and HUVEC cells. Concentration-dependent effect of MPT0E028 and SAHA on cell growth in (A) MDAMB231, (B) NCI-ADR, and (C) HUVEC cells. Cells were incubated without or with the indicated concentrations of MPT0E028 or SAHA for 48 h. Cell growth was evaluated by SRB and crystal violet assay. Data were expressed as mean ± S.E.M. of at least 3 independent experiments.
