# Supplementary material for: Anticancer Activity of MPT0E028, a Novel Potent Histone Deacetylase Inhibitor, in Human Colorectal Cancer HCT116 Cells In Vitro and In Vivo
Source: PLoS One. 2012 Aug 22;7(8):e43645. doi: 10.1371/journal.pone.0043645 (PMC3425516; doi:10.1371/journal.pone.0043645)
Supplement: Data S3 — Inhibition of total HDAC activity by MPT0E028 and SAHA in MDAMB231 and NCI-ADR cells. (A) MDAMB231 and (B) NCI-ADR cells were treated with the indicated concentrations of MPT0E028 and SAHA for 24 h, and the total lysate were subjected to total HDAC enzyme activity detection. Data are expressed as the mean±S.E.M. of at least 3 independent experiments. (PDF) [file pone.0043645.s003.pdf]

Supplemental data 3

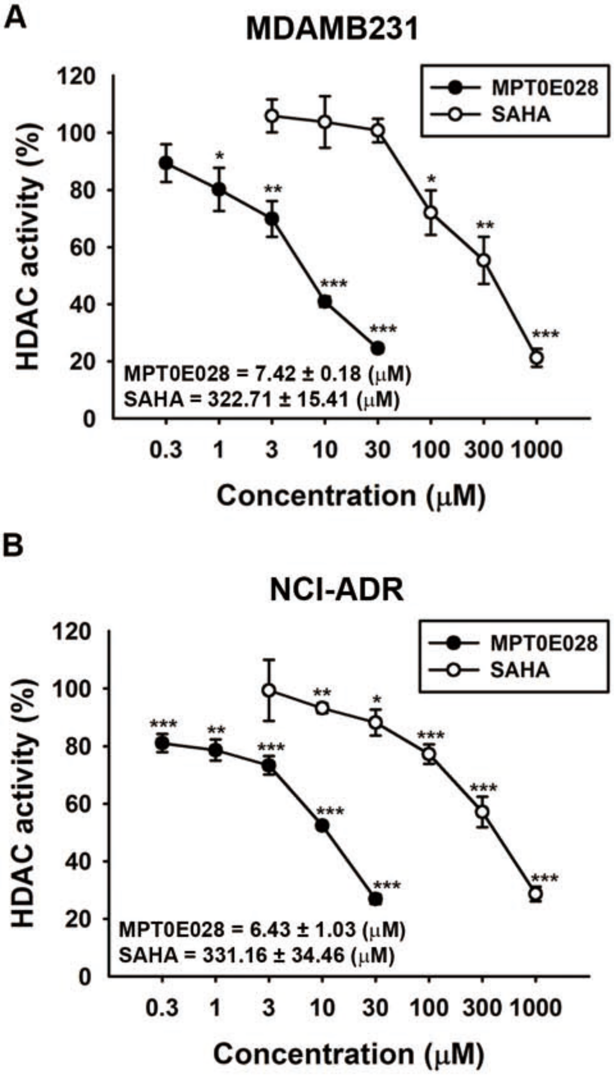

Supplemental data 3. Inhibition of total HDAC activity by MPT0E028 and SAHA in MDAMB231 and NCI-ADR cells.(A) MDAMB231 and (B) NCI-ADR cells were treated with the indicated concentrations of MPT0E028 and SAHA for 24 h, and the total lysate were subjected to total HDAC enzyme activity detection. Data are expressed as the mean ± S.E.M. of at least 3 independent experiments.
